# Editorial: Immunomodulatory roles of fibroblast-like synoviocytes in rheumatoid arthritis

**DOI:** 10.3389/fimmu.2024.1415672

**Published:** 2024-05-08

**Authors:** Yunhui Chen, Ting Han, Zhenhong Guo, Xiaoli Li, Xiaofei Zhang, Wei Peng

**Affiliations:** ^1^ School of Basic Medical Sciences, CDUTCM-KEELE Joint Health and Medical Sciences Institute, School of Pharmacy, Chengdu University of Traditional Chinese Medicine, Chengdu, China; ^2^ School of Pharmacy, Naval Medical University, Shanghai, China; ^3^ Karolinska Institute Hospital, Karolinska Institute, Huddinge, Sweden; ^4^ School of Pharmacy, Shannxi University of Chinese Medicine, Xianyang, China

**Keywords:** immunomodulatory role, fibroblast-like synoviocytes, rheumatoid arthritis, FLS, RA

Rheumatoid arthritis (RA), also known as “deathless cancer”, is a chronic and debilitating autoimmune disease that causes joint deformity and even loss of joint function with excessive synovial tissue hyperplasia, pannus formation, and cartilage erosion. The excessive proliferation of fibroblast-like synoviocytes (FLS) has been recognized as the primary pathological foundation of RA, and activated FLS can secrete various cytokines, chemokines, and metalloproteinases (MMPs) that lead to inflammation, angiogenesis, cartilage degradation, and joint damage. Thus, it plays a pivotal immunomodulatory role in RA development. This Research Topic, “*Immunomodulatory roles of fibroblast-like synoviocytes in rheumatoid arthritis*“, has provided an academic platform to decipher the immunomodulatory roles of FLS in RA development and to explore the potential molecular targets and therapeutic interventions for modulating FLS to manage RA.

The original research by Ryu et al. reported the anti-arthritic effects and underlying mechanisms of photobiomodulation (PBM) on FLS from RA patients and collagen-induced arthritis (CIA) mouse models. Rahat et al. continued their previous study to further investigate whether CD147 influences anti-angiogenic factors and revealed that CD147 secretion may regulate allosteric effects on matrix metallopeptidase 9 and proteasome 20S activities, potentially acting as a switch to turn angiogenesis on or off during RA progression. Moreover, Kong et al. explored the *in vivo* and *in vitro* effects of osteoarthritis (OA) fibroblast-like synoviocyte (OA-FLS) exosomal microRNA (miR) -19b-3p on OA ferroptosis and its potential mechanisms using OA cellular and rat models. In addition, two comprehensive and informative reviews were presented to elucidate the recent advances in this field. Wang et al. summarized the fibroblast-activated protein-a (FAP)-mediated functional phenotypic changes in FLS and their regulatory mechanisms, providing insights into RA pathogenesis and identifying potential therapeutic targets. Another review by Hu et al. illustrated the currently available evidence of metabolic changes of FLS in RA, analyzed the mechanisms of these metabolic alterations, assessed their effects on the RA phenotype, and highlighted the prospects and challenges of targeting FLS metabolism as a therapeutic strategy for RA.

This research compendium, which includes *in vivo* and *in vitro* experimental research and state-of-the-art reviews, has provided us with valuable insights to delve deeper into the immunomodulatory roles, functions, and mechanisms of FLS in RA development, in addition to its potential molecular targets and therapeutic interventions. However, several aspects remain unexplored. More original research is still warranted to uncover novel molecular mechanisms and signaling pathways related to FLS in RA development, to investigate innovative intervention strategies targeting FLS, such as apoptosis, autophagy, ferroptosis, and intestinal microflora regulation, and to identify novel molecular targets related to FLS for RA management and their potential agonists/inhibitors.

## Author contributions

YC: Conceptualization, Writing – original draft. TH: Formal analysis, Writing – original draft. ZG: Conceptualization, Formal analysis, Writing – review & editing. XL: Formal analysis, Supervision, Validation, Writing – review & editing. XZ: Formal analysis, Supervision, Writing – original draft. WP: Conceptualization, Writing – review & editing.

